# CRISPR-Enhanced Colorimetric Aptasensor for Adenosine Triphosphate Detection Based on MoS_2_-Based Nanozymes

**DOI:** 10.3390/bios15100651

**Published:** 2025-10-01

**Authors:** Zhiqiang Zhu, Haojie Ma, Huashan Yao, Yuan Yuan, Xiangyang Miao, Shao Su

**Affiliations:** 1 School of Biomedical Sciences, Suzhou Chien-Shiung Institute of Technology, Suzhou 215411, China; zhuzq@csit.edu.cn (Z.Z.); yaohs@csit.edu.cn (H.Y.); 240305711114@csit.edu.cn (Y.Y.); miaoxy@csit.edu.cn (X.M.); 2 State Key Laboratory of Flexible Electronics (LoFE) & Jiangsu Key Laboratory of Smart Biomaterials and Theranostic Technology, Institute of Advanced Materials (IAM), Nanjing University of Posts and Telecommunications, 9 Wenyuan Road, Nanjing 210023, China; 1022233718@njupt.edu.cn

**Keywords:** colorimetric aptasensor, ATP, CRISPR/Cas12a, molybdenum disulfide, nanozymes

## Abstract

As the direct energy source in organisms, accurate and simple detection of adenosine triphosphate (ATP) is of great significance. Herein, a colorimetric aptasensor for ATP determination was designed by integrating the CRISPR/Cas12a system with an aptamer, and with Prussian blue nanocube and gold nanoparticle co-functionalized MoS_2_ (MoS_2_-PBNCs-AuNPs) nanozymes. As expected, the introduced CRISPR/Cas12a system and aptamer could efficiently amplify the detection signal and improve the specific recognition ability, respectively. Meanwhile, the catalytic activity of the MoS_2_-PBNCs-AuNPs nanozymes can be regulated with the concentration of ATP. The high-affinity binding of ATP to the aptamer competitively inhibited aptamer-crRNA hybridization, causing fewer Cas12 proteins to be activated. As a result, the uncleaved single-stranded DNA (ssDNA) adsorbed onto the surface of nanozymes to effectively enhance their catalytic oxidation capability toward 3,3′,5,5′-tetramethylbenzidine (TMB). According to this phenomenon, this CRISPR-enhanced colorimetric aptasensor can detect down to 0.14 μM ATP with high selectivity, reproducibility, and stability. In addition, acceptable recoveries and low relative standard deviations of the aptasensor for ATP determination suggest that it is promising for application in early detection of clinical-related diseases.

## 1. Introduction

Adenosine triphosphate (ATP), as the primary energy currency in living organisms, powers essential cellular processes including DNA synthesis, glycolysis, transmembrane transport, and neural signaling [1,2,3]. Abnormal ATP metabolism is often associated with the occurrence of diseases, such as Parkinson’s syndrome, inflammation, hypoglycemia, and Alzheimer’s disease [4,5,6,7,8]. Therefore, quantifying the ATP level in biological fluids plays a vital role in biochemical study and clinical diagnosis. Currently, sensors have been coupled with analytical methods such as high-performance liquid chromatography (HPLC) [9], mass spectrometry [10,11,12], fluorescence assays [13,14,15], electrochemistry [16,17,18], and colorimetry to determine ATP. Among these, colorimetric sensors have been considered as a promising method for ATP detection due to their simplicity, low cost, and not needing expensive equipment. There has been a rapid development of nanozymes; the introduction of nanozymes has greatly improved the sensitivity, stability, and reproducibility of colorimetric sensors, which enlarges their practical application in ATP detection.

Since clustered regularly interspaced short palindromic repeats (CRISPR)/CRISPR-associated (Cas) systems were introduced to construct biosensors for virus detection, they have attracted significant interest in the field of sensors/biosensors [19,20,21]. In general, the activated Cas proteins can indiscriminately cleave surrounding DNA/RNA signal probes, resulting in a change in the detection signal. Coupling with the colorimetry method, the CRISPR/Cas system has been widely used to detect metal ions [22,23], small biomolecules [24,25], RNA/DNA [26,27,28], proteins [29,30], viruses [31,32], and bacteria [33,34], with exciting performance. For example, Zhao et al. developed a multicolor sensing platform for breast cancer 1 gene (BRCA1) detection by coupling with the CRISPR/Cas12a system and LSPR property of gold nanobipyramids (AuNBPs). Utilizing the etching effect of horseradish peroxidase (HRP) towards AuNBPs, the designed sensor can detect down to 30 pM BRCA1 within 60 min with high selectivity [35]. Yang and co-workers reported a dual-mode biosensor to analyze the complementary DNA of the spike protein gene (S-cDNA) by coupling with the advantages of the CRISPR/Cas12a system and upconversion nanozymes [36]. As expected, the designed biosensor can detect 320 fM and 28.4 pM ATP in luminescence mode and colorimetric mode, respectively, with the help of the CRISPR/Cas system.

**Scheme 1:**
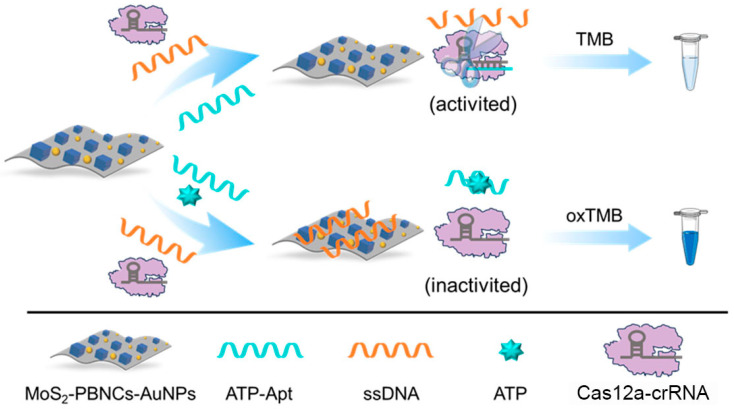
Schematic illustration of the CRISPR-enhanced colorimetric aptasensor for ATP detection based on MoS_2_-based nanozymes.

Nanozymes, especially Prussian blue-based nanozymes, have been widely used in the development of colorimetric biosensors for detecting target molecules due to their outstanding catalytic properties and excellent chemical stability [37,38,39]. Inspired by the above exciting works, a CRISPR-enhanced colorimetric aptasensor was developed for the detection of ATP by coupling, with the advantages of aptames and MoS_2_-based nanozymes. As shown in Scheme 1, a Prussian blue nanocube and gold nanoparticle co-functionalized molybdenum disulfide (MoS_2_-PBNCs-AuNPs) nanozyme with high peroxidase-like activity was synthesized; and it was employed to catalyze 3,3′,5,5′-tetramethylbenzidine (TMB) in the presence of hydrogen peroxide (H_2_O_2_). Correspondingly, an obvious colorimetric signal was generated. Importantly, the catalytic performance of MoS_2_-PBNCs-AuNPs nanozymes was enhanced with the adsorption of single-stranded DNA (ssDNA). In the absence of ATP, ATP aptamer (ATP-Apt) hybridized with crRNA to activate the cleave activity of the Cas12a protein. As a result, the adsorbed ssDNA was cleaved to small fragments by Cas12a, leading to the desorption of ssDNA from the nanozyme surface. Correspondingly, a light-blue solution color and low absorption peak were observed due to the decrease in the catalytic activity of the nanozymes. In the presence of ATP, ATP competitively binds to ATP-Apt due to the high affinity, causing less Cas12a to be activated. Obviously, a blue solution and high absorption peak, located at 652 nm, were obtained. According to this concept, the CRISPR-enhanced colorimetric aptasensor can qualitatively and quantitatively detect ATP with high performance. Moreover, the designed aptasensor can efficiently analyze ATP in real samples.

## 2. Materials and Methods

### 2.1. Materials and Reagents

Molybdenum(IV) sulfide (MoS_2_, <2 mm, 99%), n-butyllithium, chloroauric acid trihydrate (HAuCl_4_·3H_2_O, ≥99%), 3,3′,5,5′-tetramethylbenzidine (TMB), adenosine 5′-triphosphate (ATP), adenosine 5′-diphosphate (ADP), adenosine 5′-monophosphate (AMP), guanosine 5′-triphosphate (GTP), and cytidine 5′-triphosphate (CTP) were purchased from Sigma-Aldrich (Shanghai, China). Poly(*N*-vinyl-2-pyrrolidone) (PVP, MW = 58,000), potassium chloride (KCl), potassium ferricyanide (K_3_[Fe(CN)_6_], ≥99.5%), iron(III) chloride (FeCl_3_), magnesium chloride (MgCl_2_), sodium acetate (NaAc), glacial acetic acid (HAc), and 30% hydrogen peroxide (H_2_O_2_) were purchased from Sinopharm Chemical Reagent Co., Ltd. (Shanghai, China). Cas12a protein was supplied by Nanjing Vazyme Biotech Co., Ltd. (Nanjing, China). Tris-HCl buffer (1 M, pH 7.5) and DEPC-treated water were purchased from Sangon Biotech (Shanghai, China). ATP-Apt, crRNA, and ssDNA were also synthesized by Sangon Biotech (Shanghai, China); these were recorded as ACCTGGGGGAGTATTGCGGAGGAAGGT, UAAUUUCUACUAAGUGUAGAUCCUCCGCAAUACUCCCCCAGGU, and TTTTTTTTTTTTTTTTTTTTTTTTTTTTTT, respectively. All aqueous solutions were prepared with twice-deionized water.

### 2.2. Apparatus

A zeta potential analyzer (Brookhaven Instruments, New York, NY, USA) was used to characterized the different nanomaterials. The UV–vis absorption spectrum was recorded on a Shimadzu UV-3600 spectrophotometer (Tokyo, Japan).

### 2.3. Preparation of MoS_2_-Based Nanoenzymes

MoS_2_-based nanozymes were synthesized according to our previous works [38,39]. Briefly, 0.025 mg mL^−1^ exfoliated MoS_2_ solution was mixed with PVP, 40 mM K_3_[Fe(CN)_6_], and 2 M KCl at pH 1.5. Subsequently, 40 mM FeCl_3_ was added to the above mixed solution and reacted for 30 min at 60 °C to obtain MoS_2_-PBNCs nanozymes under microwave-assisted conditions. Then, 10 mM HAuCl_4_ was added dropwise to the purified MoS_2_-PBNCs solution under stirring conditions for 30 min. Finally, MoS_2_-PBNCs-AuNPs nanozymes were purified by centrifugation and stored at 4 °C in a refrigerator. Transmission electron microscope (TEM) characterization of the MoS_2_ nanosheet, MoS_2_-PBNCs, and MoS_2_-PBNCs-AuNPs was performed on a Hitachi H-7500 instrument (Tokyo, Japan).

### 2.4. Preparation of the CRISPR-Enhanced Colorimetric Biosensor

At first, a mixture solution containing 100 nM Cas12a, 100 nM crRNA, 100 nM ATP-Apt, and 500 nM ss DNA was prepared at 37 °C for 30 min. In the absence of ATP, MoS_2_-PBNCs-AuNPs nanozymes were added to the mixture solution and left to react for 15 min. After the reaction, 5 μL of 0.5 mM TMB, 10 μL of 10 mM H_2_O_2_, and 25 μL of acetate buffer (pH 4.0) were mixed with the above solution and incubated for 6 min at room temperature. Finally, the solution color and absorption peak intensity at 562 nm were recorded to evaluate the analytical performance. In the presence of ATP, the same process was performed to obtain the solution color and absorption peak intensity.

## 3. Results and Discussion

### 3.1. Characterization of MoS_2_-PBNCs-AuNPs Nanozymes

**Figure 1:**
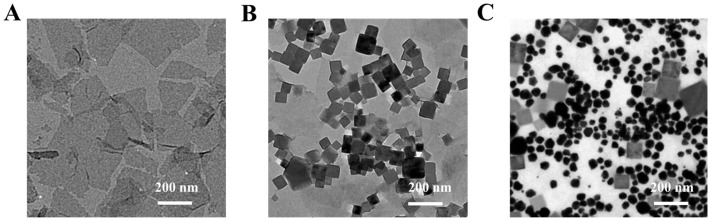
TEM images of (**A**) MoS_2_, (**B**) MoS_2_-PBNCs and (**C**) MoS_2_-PBNCs-AuNPs.

The morphologies of the different nanomaterials are shown in Figure 1. Obviously, the intercalated MoS_2_ nanosheet showed a typical layered nanostructure; it was used to prepare different nanozymes. With the assistance of microwaves, about 50 nm of PBNCs was successfully supported on the surface of the MoS_2_ nanosheet to form MoS_2_-PBNCs nanocomposites [38]. With the addition of HAuCl_4_, AuNPs and PBNCs were co-functionalized on the surface of the MoS_2_ nanosheet, forming the expected MoS_2_-PBNCs-AuNPs nanozymes.

### 3.2. ssDNA-Enhanced Catalytic Activity of MoS_2_-PBNCs-AuNPs Nanozymes

**Figure 2:**
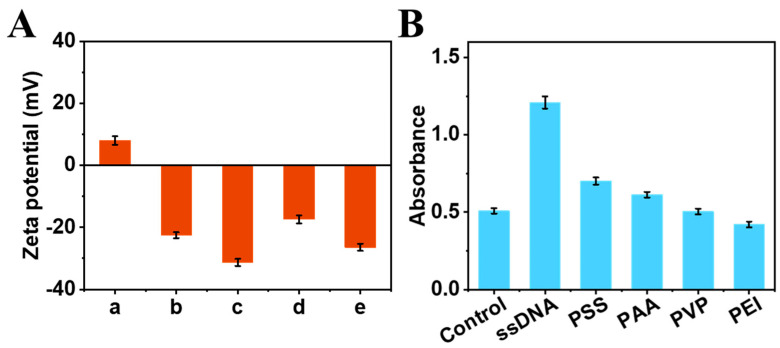
(**A**) Zeta potentials of (a) TMB, (b) MoS_2_-PBNCs-AuNPs, (c) ssDNA/MoS_2_-PBNCs-AuNPs, (d) MoS_2_-PBNCs-AuNPs + TMB, and (e) ssDNA/MoS_2_-PBNCs-AuNPs + TMB in 0.2 M acetate buffer (pH 4.0). (**B**) Peroxidase-like activities of MoS_2_-PBNCs-AuNPs nanozymes functionalized with different molecules.

The enhancing effect of ssDNA on the catalytic activity of the MoS_2_-PBNCs-AuNPs nanozymes was studied. As shown in Figure 2A, the zeta potential of MoS_2_-PBNCs-AuNPs (column b) shifted from −22.50 mV to −31.29 mV with the addition of ssDNA (column c), confirming the successful adsorption of ssDNA onto the nanozyme. It is noted that TMB carries a positive charge (+8.03 mV, column a) in acetate buffer (pH 4.0), which can adsorb onto the surface of MoS_2_-PBNCs-AuNPs nanozymes (column d) and ssDNA-functionalized MoS_2_-PBNCs-AuNPs nanozymes (column e) via electrostatic interaction. Obviously, ssDNA/MoS_2_-PBNCs-AuNPs nanozymes have more negative charges, meaning they can adsorb the more positively charged TMB substrate to generate higher catalytic performance. To better understand the effect of surface charge on the catalytic activity of MoS_2_-PBNCs-AuNPs nanozymes, we compared ssDNA with other polymers, including polystyrene sulfonate (PSS), polyacrylic acid (PAA), polyvinylpyrrolidone (PVP), and polyetherimide (PEI). Figure 2B shows that MoS_2_-PBNCs-AuNPs nanozymes functionalized with negatively charged molecules (ssDNA, PSS, PAA) exhibited enhanced catalytic performance. Meanwhile, MoS_2_-PBNCs-AuNPs nanozymes decorated with positively charged PEI showed a decrease in catalytic performance. Interestingly, the catalytic activity of uncharged PVP functionalized MoS_2_-PBNCs-AuNPs nanozymes was almost the same as MoS_2_-PBNCs-AuNPs nanozymes. It should be noted that ssDNA-functionalized MoS_2_-PBNCs-AuNPs nanozymes showed the best catalytic activity toward TMB oxidation in this work.

### 3.3. Detection Feasibility of CRISPR-Enhanced Colorimetric Aptasensor

**Figure 3:**
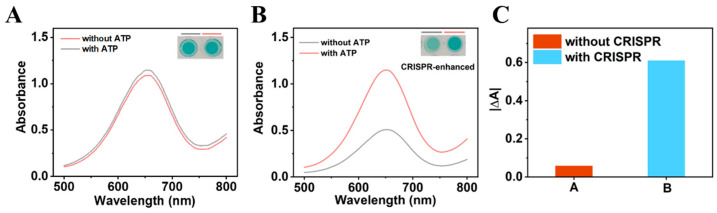
(**A**) UV–vis absorption spectra and photo (inset) of a conventional colorimetric aptasensor for ATP detection based on MoS_2_-PBNCs-AuNPs nanozymes. (**B**) UV–vis absorption spectra and photo (inset) of a CRISPR-enhanced colorimetric aptasensor for ATP detection based on MoS_2_-PBNCs-AuNPs nanozymes. (**C**) The performance change of colorimetric aptasensors for ATP detection with CRISPR-enhancement and without CRISPR-enhancement.

To evaluate the signal amplification effect of the CRISPR/Cas system, a common colorimetric aptasensor was designed based on MoS_2_-PBNCs-AuNPs nanozymes. As shown in Figure 3A, the addition of ATP resulted in only a minor change in the absorption peak intensity of the colorimetric aptasensor, which was ascribed to the conformational change in ATP-Apt. The ATP-Apt complex formed may be also adsorbed on the surface of nanozymes, leading to a minor increase in absorption peak intensity. As expected, there was an obvious change in the absorption peak intensity of the CRISPR-enhanced colorimetric aptasensor with the addition of ATP (Figure 3B). The reason is that the activated Cas12a protein efficiently cleaved the ssDNA, leading to fragmented ssDNA being unable to adsorb onto the surface of the nanozymes. Consequently, the absorption peak intensity change (∆A = A_ATP_ − A_no ATP_) of the CRISPR-enhanced colorimetric aptasensor is about 10.5 times higher than that of the conventional colorimetric aptasensor for 5 μM ATP detection (Figure 3C). All the experimental results suggested that the CRISPR-enhanced colorimetric aptasensor is a promising sensing platform for ATP detection.

### 3.4. Optimization of Experimental Conditions

**Figure 4:**
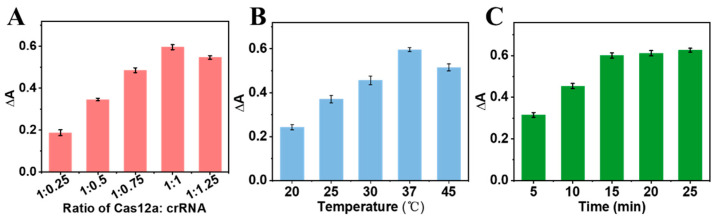
The effects of (**A**) the Cas12a and crRNA molar ratio, (**B**) CRISPR/Cas system reaction temperature, and (**C**) CRISPR/Cas system reaction time on the sensing performance of the CRISPR-enhanced colorimetric aptasensor for ATP detection.

To obtain the best sensing performance, the effects of different experimental conditions on the CRISPR-enhanced colorimetric aptasensor were investigated. At first, the molar ratio of the Cas12a protein and crRNA was studied. It can be found that ∆A of the colorimetric aptasensor increased with an increasing ratio of the Cas12a protein and crRNA. The largest ∆A was obtained when the ratio of the Cas12a protein and crRNA reached 1:1 (Figure 4A). Therefore, the same molar concentrations of Cas12a and crRNA was selected as the optimal condition. Similarly, the maximum ∆A was obtained when the reaction temperature was 37 °C (Figure 4B). If the reaction temperature was up to 45 °C, the ∆A of the aptasensor decreased. This is ascribed to the high temperature affecting the catalytic activity of the Cas12a protein. Therefore, the optimal reaction temperature was chosen as 37 °C. Finally, the incubation time of the CRISPR/Cas system was optimized. As shown in Figure 4C, the ∆A of the aptasensor increased with increasing the incubation time over the range of 0–15 min. There was almost no change in ∆A when the reaction time was prolonged to 25 min. To save on the detection time, the optimal incubation time of CRISPR/Cas system was selected as 15 min.

### 3.5. Analytical Performance of the CRISPR-Enhanced Colorimetric Aptasensor

**Figure 5:**
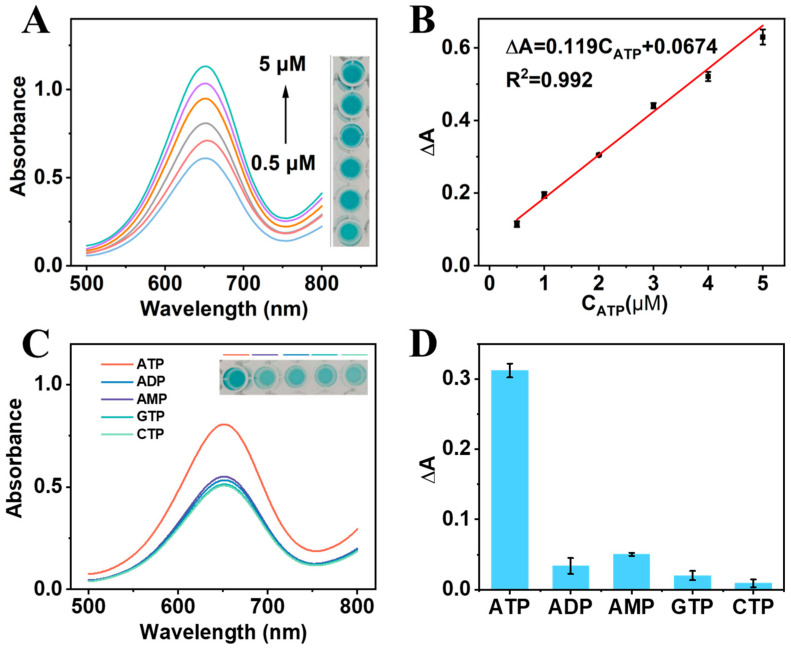
(**A**) UV–vis absorption spectra and photos of the CRISPR-enhanced colorimetric aptasensor in the presence of ATP at increasing concentrations (0.5, 1, 2, 3, 4, and 5 μM). (**B**) The linear relationship of ΔA and ATP concentrations. (**C**) UV–vis curves of the aptasensor for ATP, ADP, AMP, GTP, and CTP detection. Inset: The corresponding solution color of the aptasensor for different molecules detection. (**D**) The ΔA of the aptasensor for ATP, ADP, AMP, GTP, and CTP detection.

The performance of the CRISPR-enhanced colorimetric aptasensor for ATP detection was investigated under the optimal conditions. Obviously, a higher ATP concentration resulted in a larger increase in the absorption peak intensity of the CRISPR-enhanced colorimetric aptasensor (Figure 5A). The corresponding solution color also proved this result (inset in Figure 5A). The added ATP competitively bound to ATP-Apt, resulting in a reduction in ATP-Apt bound to crRNA. Correspondingly, fewer Cas12a proteins were activated and less ssDNA was cleaved. Consequently, more ssDNA adsorbed onto the surface of nanozymes, enhancing their catalytic activity. As shown in Figure 5B, a linear range between the ∆A of the CRISPR-enhanced aptasensor and 0.5–5 μM ATP was obtained. The linear equation was obtained as ∆A = 0.119C_ATP_ + 0.0674, with a correlation coefficient of 0.992. According to the slope of the equation and standard deviation of the blank samples, the detection limit (LOD) was calculated to be 0.14 μM (S/N = 3), which was comparable to or better than in other published works (Table 1).

To study the selectivity of the CRISPR-enhanced colorimetric apatsensor, adenosine diphosphate (ADP), adenosine monophosphate (AMP), cytidine triphosphate (CTP), and guanosine triphosphate (GTP) were selected as interfering substances. As shown in Figure 5C, the same concentrations of ADP, AMP, CTP, and GTP do not cause an obvious change in the absorption peak intensity. The ∆A of the CRISPR-enhanced colorimetric aptasensor for ATP detection was about 9 times, 6 times, 16 times, and 35 times higher than that for ADP, AMP, CTP, and GTP detection, respectively, indicating that the aptasensor has excellent selectivity (Figure 5D).

### 3.6. Reproducibility, Stability, and Practical Applicability of This CRISPR-Enhanced Aptasensor

**Figure 6:**
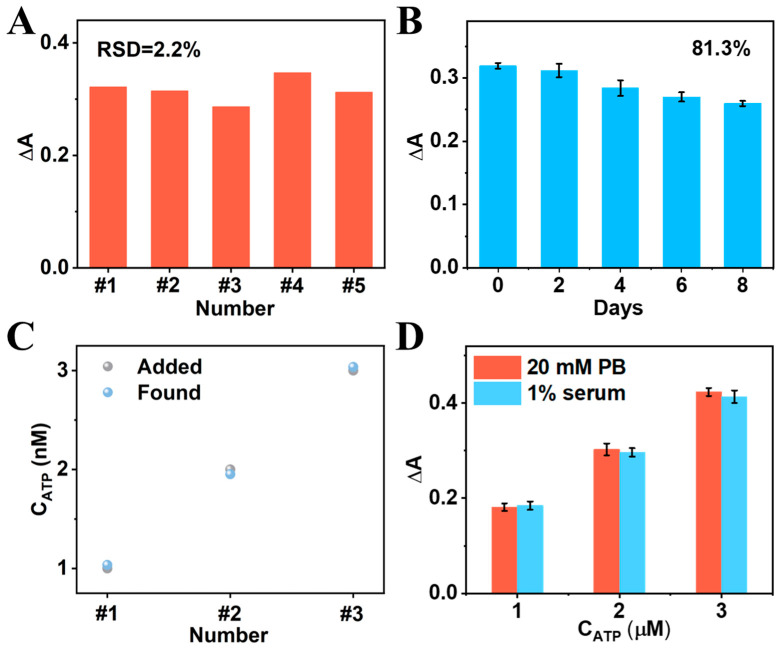
(**A**) Reproducibility and (**B**) stability of the CRISPR-enhanced colorimetric aptasensor. (**C**) The performance of the aptasensor for different concentrations of ATP (1 μM, 2 μM, 3 μM) in 1% serum by using standard addition method. (**D**) Comparison of the aptasensor for different concentrations of ATP (1 μM, 2 μM, 3 μM) detection in buffer and in 1% serum.

At first, five independent CRISPR-enhanced colorimetric aptasensors were developed to study their reproducibility. The relative standard deviation (RSD) of the five aptasensors for the same concentration of ATP detection was only 2.2%, proving that the designed aptasensor has excellent reproducibility (Figure 6A). After 8 days of storage, the ΔA of the aptasensor was still 81.3% of the initial value (Figure 6B), suggesting that the aptasensor has good storage stability. Subsequently, the standard addition method was used to evaluate the practical application of this CRISPR-enhanced colorimetric aptasensor. As shown in Figure 6C, the recoveries and RSD of the aptasensor for detecting clinically relevant ATP concentrations (1 μM, 2 μM, and 3 μM) in serum were about 95.06–99.50% and 3.53–6.16%, respectively, indicating that the designed colorimetric aptasensor is promising for practical application. In addition, the ΔA of the aptasensor for the same concentrations of ATP detection in 1% serum samples and in ideal PB buffer are almost the same, further suggesting that the developed aptasensor has good anti-interference ability and potential for application in clinical samples (Figure 6D).

## 4. Conclusions

In summary, a colorimetric aptasensor was successfully developed to sensitively and selectively detect ATP by coupling with the advantages of the CRISPR/Cas system, an aptamer, and MoS_2_-PBNCs-AuNPs nanozymes. The added ATP can efficiently regulate the number of activated Cas12a proteins, thereby regulating the enhancement effect of ssDNA on MoS_2_-PBNCs-AuNPs nanozyme activity. Due to the signal being amplified by the CRISPR/Cas system, the designed colorimetric aptasensor exhibited a wide linear range (0.5–5 μM), a low detection limit (0.14 μM), high selectivity, and excellent reproducibility for ATP detection. With its validated detection performance, this aptasensor showed promising applicability for early-stage screening of disease biomarkers.

## Figures and Tables

**Table 1:** Comparison of the performances of different colorimetric sensors for the determination of ATP.

Sensor	Linear Range (μM)	Limit of Detection (μM)	Ref.
Carbon dot-based sensor	0.05–2 μM	0.034 μM	[40]
Chlorophosphonazo III-Mg^2+^ complex-based sensor	0–30 μM	1.55 μM	[41]
Quercetin@ZIF-90-based sensor	2–80 μM	0.58 μM	[42]
Zeolitic imidazolate framework-based sensor	10–240 μM	3.85 μM	[43]
CRISPR-mediated sensor	5–50 μM	2.5 μM	[24]
CRISPR-enhanced aptasensor	0.5–5 μM	0.14 μM	This work

## Data Availability

The datasets generated during the current study are available from the corresponding author on reasonable request.
